# Influence of Lameness on the Lying Behaviour of Zero-Grazed Lactating Jersey Dairy Cattle Housed in Straw Yards

**DOI:** 10.3390/ani9100829

**Published:** 2019-10-19

**Authors:** Nicola Blackie, Lawrence Maclaurin

**Affiliations:** 1 Pathobiology and Production Sciences, Animal Welfare Science and Ethics, Royal Veterinary College, Hawkshead Lane, Hatfield AL9 7TA, Hertfordshire, UK; 2 Centre for Equine and Animal Science, Writtle University College, Chelmsford CM1 3RR, UK

**Keywords:** Jersey, dairy cattle, locomotion score, lying behaviour, lameness, automatic behaviour monitoring, prevalence, zero-grazing

## Abstract

**Simple Summary:**

Lameness is a concern within the dairy industry. Firstly, it has a negative effect on the animal’s welfare, and secondly, has been found to be costly to producers. Ultimately, it can result in having to cull the cow early. Lameness can change the behaviour of cows who may spend more time lying down to relieve the pain in their feet. In this study, we used activity monitors to automatically record the behaviour of non-lame and lame Jersey cattle. Unlike previous studies involving other breeds, the Jersey cows in this study showed no statistically significant differences in the length of time spent lying or standing in relation to lameness status. Lame cows did have significantly shorter average lying bouts compared to non-lame cows. We also recorded what proportion of the herd were lame. The results (38%) were higher than expected in Jersey cattle but may be related to the fact the cows were housed all year round and therefore were not given access to grass. In conclusion, we saw no evidence of an effect of lameness on lying behaviour in this study.

**Abstract:**

Thirty-five lactating Jersey cows were recruited to the study. They were grouped according to locomotion score (LS), where low scores indicate normal gait. LS-1 (*n* = 12), LS-2 (*n* = 12) and LS-3 (*n* = 11) were used. Locomotion scores were balanced for parity and stage of lactation. Lying behaviour was recorded using IceTag™ data loggers attached to the cows for four consecutive days. The study animals remained in the straw based yards with grooved concrete flooring throughout the duration of the study. All data were normally distributed and assessed using a one-way ANOVA with a post hoc Tukey test. There were no statistically significant differences between locomotion score and the time spent lying, active and standing of zero-grazed lactating Jersey dairy cattle housed on straw yards. Lame cows (LS-3) had significantly shorter lying bouts than sound cows (LS-1) (34 min vs. 42 min, respectively). There has been limited research to date measuring the lying behaviour of cattle on straw and into the Jersey breed. The cows had longer than expected standing times and an increased frequency of lying bouts. This may have been attributed to the stocking density in which the cows were kept. We also reported a prevalence of lameness within the herd of 38%.

## 1. Introduction

Over the last twenty years, the UK herd size has been increasing from an average herd size of 80 (1998) to 151 in 2018 [1]. This increase in herd size can lead to more cows per stockperson taking care of them. As a result, time for tasks such as lameness detection may be limited. In addition, lameness prevalence in the UK has become higher in that time frame. Prevalence of lameness in the UK in the early 1990s was around 17%–20% (range 2%–54%) [2,3]. A recent study conducted in England and Wales on 61 herds showed the prevalence of lameness to be 31.6% (5.8%–65.4%) [4]. A further UK study estimates lameness prevalence of 30.1% (7.3%–60.6%) in 43 herds [5]. These studies indicate that prevalence has only improved marginally since 2007, where the prevalence of lameness was 36.8% (0%–79.2%) [6]. Access to pasture can impact lameness with zero-grazed cattle having higher prevalence of lameness [7] and access to pasture improving lameness scores [8]. Straw yards are also associated with a lower prevalence of lameness [4,6,9]. Visual mobility scoring is the most commonly implemented tool when assessing the prevalence and incidence of lameness. As visual mobility scoring is time consuming, it may be more favourable to utilise automatic lameness detection systems which can identify changes in an animal’s behaviour automatically [10].

Not only does lameness reduce productivity in terms of milk production [11,12,13,14], it can affect fertility [15,16] and the welfare of dairy cattle [17]. Lameness can also affect the behaviour of dairy cows [18], resulting in disruptions to their activity budgets [19] and oestrus expression [20,21,22]. These changes in activity budget could be used to detect lameness. Lame cows spend more time lying down in most [19,21,23,24,25,26,27,28], but not all, studies [29]. They also have longer lying bouts [19,24,25,28,30], spend less time feeding [26,31,32,33] and may come into the milking parlour later [34,35].

The ability to spend time lying down is extremely important to the welfare of dairy cows. Indeed, cows are highly motivated to perform this behaviour [36], particularly for access to deep bedding [37].

The majority of data available on the prevalence/incidence of lameness and behaviour of lame cows is based on the Holstein breed. Jersey cattle are less studied despite them being the second most common dairy breed worldwide. One study showed Jersey and crossbred cattle to have a lower incidence of lameness than Holstein Friesian in New Zealand [38]. Although, the incidence of lameness was low in the aforementioned study.

The aim of this study is to investigate the impact of lameness on the lying behaviour of zero-grazed Jersey cattle within a loose housed system.

## 2. Materials and Methods

### 2.1. Animals and Housing

The study was conducted on a 525 cow, zero-grazed, straw yard housed dairy herd in the south east of the UK. Thirty-five Jersey cows (29 primiparous and 6 multiparous) were recruited. The study was approved by the Writtle College Animal Welfare Ethics Committee (98331793-AW1).

The cows were housed all year round and received fresh straw daily (07:00 to 08:00). Cows received a total mixed ration once daily, offered between 08:00 and 09:00, which was presented in troughs. The ration was made up of maize, silage, grass silage, concentrate, molasses with urea supplement, crimped wheat and brewer’s grains. The cows were housed in a loose yard which was divided into 5 pens of 105 cows; experimental animals were distributed across the groups. The lying space allocation was 4.42 m^2^/cow. Passageways were grooved concrete and scraped 2–3 times a day by a scraper tractor. The animals were milked twice daily through a rotary parlour holding 28 cows at a time; the cows were producing an average of 27.2 ± 2.47 litres (mean ± standard error) of milk per day over the period of the study, compared to the heifer average of 21.2 ± 1.16 litres of milk per day over the period of the study. Milking took place at 05:30 and 14:30 daily.

Assessment of locomotion score (LS) of the entire herd was conducted using the Agriculture and Horticulture Development Board (AHDB) mobility scoring system, as shown in Table 1. The AHDB mobility scoring is designed to detect cows for functional trimming, and scores of 0 and 1 are generally combined to represent “acceptable mobility”. After completion of the locomotion scoring, the cows were assigned to groups dependent on their score. These groups were then balanced for parity and stage of lactation. Cows who scored 0 or 1 were grouped together and named LS-1 (acceptable mobility, i.e., not lame). This was an observational study whereby we did not interfere with normal farming practices. The farm staff were given a list of cows identified as lame and they were treated as soon as possible following the study. These cows were selected based on the score prior to the start of the study and no attempts were made to ensure a cow remained in a locomotion score group, i.e., a routine foot trimming program was underway on the farm. Following assessment for mobility, thirty-five cows were recruited to the study; LS-1 (*n* = 12) parity 1.3 ± 0.22 and 222.1 ± 33.11 days in milk, LS-2 (*n* = 12) parity 1.4 ± 0.29 and 189.4 ± 38.55 days in milk and LS-3 (*n* = 11) parity 1.4 ± 0.24 and 228.3 ± 32.41 days in milk.

Prevalence of lameness was calculated from the locomotion scores obtained from the whole herd and was calculated as the proportion of the herd scoring 2 or 3, i.e., had impaired or severely impaired mobility.

### 2.2. Data Collection and Analysis

Immediately after locomotion scoring, activity monitors (IceTags™, Ice Robotics Ltd., Roslin, UK) were attached to the right hind legs of the cows just above the fetlock using a Velcro strap. The IceTags™ were attached for 5 days to record the standing and lying behaviour of the cows. Day 1 was excluded from analysis to allow the cows to adapt to the devices, however, it is noted that pervious research suggests the device does not affect lying behaviour following 14 h of habituation [39]. The data recorded from the IceTags™ were used to determine the percentage of time the cows spent standing, lying and active over 4 days. These data were then converted to hours/day to show the activity budget of the cow.

The duration of lying bouts were calculated as the number of consecutive minutes that the percentage of time spent lying was recorded as being greater than 90% (i.e., the cow had been lying down for at least 54 of the previous 60 s) [19]. The number of lying bouts per day was determined as the mean number of times a cow shifts from standing to lying during a 24 h period [19]. From the lying data, the minimum and maximum duration of lying bouts were calculated; the average of 4 days of study for each cow were analysed. The distribution of lying behaviour throughout the day was also assessed with every hour the cow was defined as standing or lying. From this information, the proportion of cows in each locomotion score group that were lying down were calculated for each hour.

The IceTag data analysed were a mean of 4 days data. All data were normally distributed (according to Pearson’s skewness test) and differences between locomotion score (LS) groups were assessed by one-way ANOVA using Genstat 16th Edition (VSN International, Hemel Hempstead, UK). Where the effects of LS were significant, Tukey’s post hoc test was used for multiple comparisons between groups. All alpha levels were set at 0.05.

## 3. Results

In our study, all lameness groups had a mean lying time of approximately 9.5 h, as shown in Table 2. The differences between the LS groups were not statistically significant, however, the standard deviation (SD) increased with increasing LS, indicating more variation. Cows with LS-3 spent 0.08 h less lying down than LS-1 cows, and 0.17 h less lying down than LS-2 cows. In addition, the differences in time spent active, time spent standing and steps/day were not statistically significant.

Average duration of lying bouts was significantly different across locomotion score groups. The average duration of lying bouts decreased as locomotion score increased (*p* = 0.017). Sound cows LS-1 had 6.8 and 7.9 min longer lying bouts than cows with LS-2 and LS-3, respectively. Maximum duration of lying bouts was slightly longer for cows with LS-1 compared to cows with LS-2 (8.6 min longer) and LS-3 (14.3 min longer), although these differences were not statistically significant (*p* = 0.294). There was a tendency for the frequency of lying bouts to be lower in the LS-1 group (13.3 bouts per day), compared to LS-2 and LS-3 groups (15.6 bouts per day and 16.0 bouts per day, respectively); this difference was not statistically significant (*p* = 0.131).

**Figure 1:**
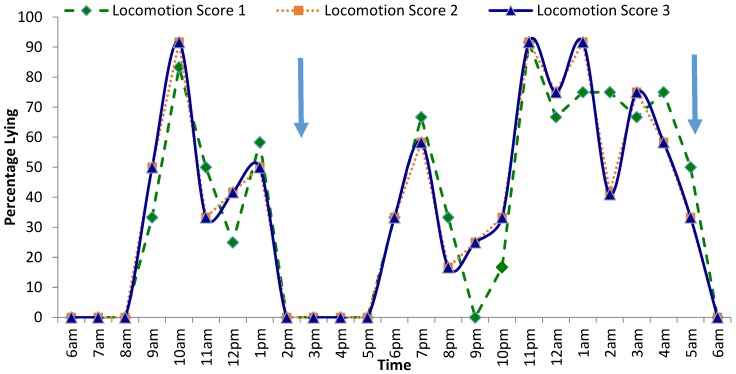
The activity of lactating Jersey dairy cattle over a 24 h period; solid arrows represent start of milking time (05:30 and 14:30). Bedding was refreshed between 07:00 and 08:00 and fresh feed offered around 08:00 into trough—feed was not pushed up.

Despite the lying times not being statistically different across locomotion score groups, differences in lying behaviour were observed at different times throughout the day. The percentage of cows lying down over a 24 h period can be seen in Figure 1. Post milking, between 09:00 and 10:00, the majority (over 90%) of LS-2 and LS-3 cows were lying down whilst fewer (83%) LS-1 cows were lying down. More LS-2 and LS-3 were recorded lying down at 12:00 than LS-1 cows. At 21:00, 0% of LS-1 cows were recorded lying down whereas 25% of LS-2 and LS-3 cows were lying down. This trend continued throughout the night with more LS-2 and LS-3 cows recorded lying down between 21:00 and 01:00. Most cows were recorded to be standing between 06:00 and 09:00 and 14:00–17:00 as this coincided with morning and afternoon milking.

The overall prevalence of lameness on the farm was 37.97%. Of the cows that were locomotion scored; 63.0% were classified LS 1, 32.8% were LS 2 and the remaining 4.2% were LS3.

## 4. Discussion

A limitation of our study was that sample size was restricted by the fact we chose to balance the groups for parity and stage of lactation to strengthen the study design. However, as cows had to meet criteria of lameness status, parity and stage of lactation, this reduced the pool of animals available for the study. Thus, although we saw no statistically or biologically important differences in some measures, further work may be needed to confirm our findings.

The findings of the present study suggest that lameness had no statistically significant effect on the time spent lying, standing and active of zero-grazed lactating Jersey dairy cattle housed on straw yards. Cows in this study had an overall mean of 9.5 h/day lying down. Non-lame cows (LS-1) were able to achieve slightly longer lying times by having a lower frequency of significantly longer mean lying bouts and slightly longer maximum lying bouts. The frequency of lying bouts in the current study was higher than the values of 8 to 11 bouts/day reported in other studies [19,40,41,42]. The cows in the present study were housed on straw. A possible hypothesis is that straw provides good support and traction, and the cows were confident they could rise and lay down without difficulty or pain, hence potentially increasing the frequency of lying bouts. The cows showed similar frequency of lying bouts as those shown on pasture [8,43] and slightly more than those seen in compost pack systems (9–12.5) [44]. The higher frequency of lying bouts may potentially be attributed to the disruptive nature of an open (loose) system. The pens were long and thin which may have caused disturbance when a cow got up to feed. It has also been shown that Jersey cattle have a tendency to eat meals more regularly throughout the day compared to Holstein cattle [45]. This may result in more transitions from lying to standing to go and feed.

It is widely reported in published literature, that a relationship exists between increased locomotion score and an increase in the lying behaviour of lame cows [19,21,23,24,25,26,27]. However, in contrast, we saw no statistical difference in time spent lying down between lame and sound cows in the present study. In order to have 80% probability of detecting a mean difference of 1 h in a population where the SD of the non-lame group was 0.94 and the SD of the lame group was 2.2, as shown in Table 2, we would have needed 45 cows in each group. Lame cows in the present study spent 9.4 h/day lying down, which is similar to the lying time of 9.7 h/day reported by Singh et al. [46]. However, the lying times recorded in the current study are much less than the 13.0–14.0 h/day lying times of straw housed cows reported by Fregonesi and Leaver [47]. Despite such a variation in lying times, Singh et al. [46] reported that for straw housed dairy cattle, lying times of 10 h/day are adequate, which is close to the results of the current study. These differences may be a result of the breed of dairy cow that were recruited to the aforementioned studies. The present study focussed on the Jersey breed whereas most other lameness studies used Holstein-Friesian cows [19,21,24,47]. However, studies focusing on the Jersey breed are scarce. A study looking at Holstein, crossbred and Jersey cattle reported an average lying time of 11.1 h/day [48], but breed differences were not available. Non-lactating Jersey cattle have higher lying times of 13 h/day indoors [49] in cubicles bedded with sand. Non-lactating Jersey crossbreeds have shown similar lying times to the present study on outdoor stand-off pads [50]. We also did not insist that cows were not foot trimmed during the study. The farm staff were free to treat any of the severely lame cows at any point. Otherwise this would raise ethical issues. If the cows were treated during the study (we do not have data on this), then this might have reduced the differences seen. Where we do see differences, these are potentially conservative estimates.

It is also possible that the differences in lying times could have been a direct result of space allowances provided (4.42 m^2^/cow) to the cattle in the current study. This is below the recommendation of 1 m^2^ per 100 kg (minimum of 5 m^2^), which is the lowest of the UK standards. This may have resulted in decreased lying times seen as the animals were slightly overstocked. Beef heifers showed reduced lying time with decreasing space allowances [51]. Where cattle have reduced space allowance or are overstocked, we see a reduction in lying time [52,53,54,55,56] and number of cows lying down [57].

Also, the current study used mainly primiparous cows, with primiparous and multiparous cows housed together throughout the study. This mixing of primiparous and multiparous cows could explain the reduction in lying times as older, more dominant cows are likely to be aggressive towards lower social rank cows, particularly heifers [58,59,60]. Primiparous cows may have disturbed lying and feeding behaviour when housed with older animals [60,61]. This theory is supported by Galindo and Broom [18], who observed that lower ranking animals had lower lying times than higher ranking herd mates (7.6 h/day compared to 8.9 h/day, respectively). Loose housing can also result in more aggression as the cows do not have cubicles to regulate lying positions and potentially to hide in. These lying times seen in the present study are particularly low and could potentially indicate a welfare issue. The farm in question made changes to the management, including increasing the space allowances, following the outcome of this study. Standing behaviour was not statistically different across locomotion score groups, with lame cows (LS-3) spending just 6 min more standing than sound cows (LS-1). Walker et al. [21] found no effect of lameness on standing behaviour between lame and non-lame cows. The study took place in the summer with ambient temperatures up to 26 °C. High temperatures may cause cattle to stand for longer periods of time so they could maximise their surface area for heat loss [62].

Large numbers of cows across all locomotion score groups were recorded lying down post-milking. Although several hours post milking around midday, it can be seen only 25% of LS-1 cows were lying down compared with 40% of LS-2 and LS-3 cows. This could indicate that the LS-1 cows were having a second feeding bout before afternoon milking. Alternatively, it could show that the LS-2 and LS-3 cows have bullied the LS-1 cows away from the lying area forcing them to stand or that the lame cows were already lying down. Fewer lame cows were lying down between 02:00 and 03:00 than non-lame cows. This may be a result of lame cows trying to feed at quieter times in an attempt to reduce the likelihood of being involved in aggressive interactions. A similar pattern was seen in the study of Blackie et al. [28]. It is also possible that as the stocking density was quite high, that cows were taking it in turns to lie down. This can be seen in studies of overstocked cows. A limitation of the current study is the sample size (*n* = 35), however, Ito et al. [40] concluded that an estimate of lying time can be made using 30 focal animals monitored over 3 days.

Anecdotally, it is thought that Jerseys may be less prone to lameness than other dairy breeds. The prevalence of lameness in the current study was 38%, which is much higher than that reported by Chawala et al. [38] who reported a prevalence of 6%. The work by Chawala et al. was based in New Zealand where the systems are predominately pasture-based, and their lameness prevalence overall was extremely low. The present study showed similar prevalence of lameness to other zero-grazed herds [7] and to other UK studies [4,5,6]. A study in Wisconsin compared a pen of Holstein cattle with a pen of Jersey and Holstein x Jersey cattle and found significantly higher lameness prevalence in the former (28.9% and 15.9%, respectively) [63]. We would expect lameness to be lower in the present study due to housing the cattle in straw yards. Straw yard housed cows generally have lower prevalence of lameness compared to cubicle housing [4,7,9,64,65]. There are very few data available on the prevalence of lameness in the Jersey breed and its risk factors. This warrants future research and consideration.

## 5. Conclusions

Although we did not have sufficient numbers of cows in this pilot study to be statistically confident, we did not observe differences in time spent lying, standing and active that were of a magnitude that would be biologically important. Lying times were shorter than expected; we hypothesize that standing times may be extended due to the comfort of straw underfoot. There may also be an impact of stocking density, with the cows in the study being slightly overstocked, leading to their lying time being limited. This may have resulted in an increased number of lying bouts and the reduced lying times. We also saw higher than expected lameness prevalence, which again may be linked to extended standing times.

## Figures and Tables

**Table 1:** Agriculture and Horticulture Development Board (AHDB) Dairy mobility scoring system.

Score Category	Locomotion Score (LS)	Description of Cow Behaviour
Good Mobility	0	Walks with even weight bearing and rhythm on all four feet, with a flat back. Long, fluid strides possible.
Imperfect Mobility	1	Steps uneven (rhythm or weight bearing) or strides shortened; affected limb or limbs not immediately identifiable.
Impaired Mobility	2	Uneven weight bearing on a limb that is immediately identifiable and/or obviously shortened strides (usually with an arch to the centre of the back).
Severely Impaired Mobility	3	Unable to walk as fast as a brisk human pace (cannot keep up with the healthy herd). Lame leg easy to identify—limping; may barely stand on lame leg/s; back arched when standing and walking. Very lame.

Source: https://dairy.ahdb.org.uk/resources-library/technical-information/health-welfare/mobility-score-instructions.

**Table 2:** The effects of locomotion score (LS) on the standing, lying and active behaviour and milk production of straw housed zero-grazed lactating Jersey dairy cattle. Mean ± SD presented in each column, overall standard error of difference (S.E.D.) also presented. LS-1 represents cows with acceptable mobility, LS-2 cows with impaired mobility and LS-3 severely impaired mobility.

Parameter Measured	LS-1 * (*n* = 12)	LS-2 (*n* = 12)	LS-3 (*n* = 11)	S.E.D.	*p*-Value
Active (hours per day)	1.16 ± 0.31	1.22 ± 0.27	1.19 ± 0.12	0.10	0.844
Lying (hours per day)	9.50 ± 0.94	9.65 ± 1.3	9.42 ± 2.2	0.63	0.933
Average lying bout length (minutes)	42.75 ^b^ ± 3.2	35.97 ^a,b^ ± 5.7	34.88 ^a^ ± 5.7	2.82	0.017
Maximum lying bout length (minutes)	124.8 ± 19.4	116.2 ± 20.9	110.5 ± 27.6	9.05	0.294
Lying bout count per day	13.31 ± 3.8	15.58 ± 7.9	16.00 ± 14.3	1.39	0.131
Standing (hours per day)	13.34 ± 0.8	13.13 ± 1.3	13.40 ± 2.1	0.61	0.900
Steps per day	3301 ± 848	3496 ± 816	3336 ± 217	295.4	0.779
Milk Yield (litres per day)	20.67 ± 5.9	23.00 ± 6.9	23.18 ± 7.1	2.75	0.595
Days in lactation	222.1 ± 115	189.4 ± 134	228.3 ± 112	50.05	0.707

* LS-1: combination of score 0 and 1 cows—defined as “acceptable mobility” therefore unlikely to require functional hoof trimming. ^a,b^ Indicate significant differences within rows *p* < 0.05. Data analysed using ANOVA and post hoc Tukey test.
